# The prevalence of rodent-borne zoonotic pathogens in the South Gobi desert region of Mongolia

**DOI:** 10.1080/20008686.2023.2270258

**Published:** 2023-10-19

**Authors:** Carol Esson, Gustaf Samelius, Tanja M. Strand, Åke Lundkvist, Johan R. Michaux, Therese Råsbäck, Tara Wahab, Tserennadmid Nadia Mijiddorj, Lee Berger, Lee F. Skerratt, Matthew Low

**Affiliations:** a One Health Research Group, Medical and Veterinary Sciences, James Cook University, Townsville, Queensland, Australia; b Snow Leopard Trust, Seattle, Washington, USA; c Nordens Ark, Åby Säteri, Hunnebostrand, Sweden; d Zoonosis Science Centre, Department of Medical Biochemistry and Microbiology, Uppsala University, Uppsala, Sweden; e National Veterinary Institute (SVA), Uppsala, Sweden; f Laboratoire de génétique de la conservation, Institut de Botanique, Université de Liège, Liège, Belgium; g Animal Sante Territoire Risque Environnement, Centre International de Recherche Agronomique pour le Developpement, Institut National de la Recherche Agronomique, Université de Montpellier, Montpellier, France; h Public Health Agency of Sweden, Stockholm, Sweden; i Snow Leopard Conservation Foundation, Ulaanbaatar, Mongolia; j One Health Research Group, Melbourne Veterinary School, University of Melbourne, Melbourne, Victoria, Australia; k Department of Ecology, Swedish University of Agricultural Sciences, Uppsala, Sweden

**Keywords:** Rodent, zoonoses, leptospira, hantavirus, haematology, Mongolia

## Abstract

The alpine ecosystems and communities of central Asia are currently undergoing large-scale ecological and socio-ecological changes likely to affect wildlife-livestock-human disease interactions and zoonosis transmission risk. However, relatively little is known about the prevalence of pathogens in this region. Between 2012 and 2015 we screened 142 rodents in Mongolia’s Gobi desert for exposure to important zoonotic and livestock pathogens. Rodent seroprevalence to *Leptospira* spp. was >1/3 of tested animals, *Toxoplasma gondii* and *Coxiella burnetii* approximately 1/8 animals, and the hantaviruses being between 1/20 (Puumala-like hantavirus) and <1/100 (Seoul-like hantavirus). Gerbils trapped inside local dwellings were one of the species seropositive to Puumala-like hantavirus, suggesting a potential zoonotic transmission pathway. Seventeen genera of zoonotic bacteria were also detected in the faeces and ticks collected from these rodents, with one tick testing positive to *Yersinia*. Our study helps provide baseline patterns of disease prevalence needed to infer potential transmission between source and target populations in this region, and to help shift the focus of epidemiological research towards understanding disease transmission among species and proactive disease mitigation strategies within a broader One Health framework.

The mountainous dry alpine ecosystems of Central Asia (hereafter High Asia) have been considered a region of low disease emergence risk [1], and consequently somewhat neglected in terms of disease research at the interface of humans, livestock and wildlife. However, a recent review highlights how large-scale regional changes in ecological, socio-ecological, and socio-economic factors are affecting wildlife-livestock-human interactions in this area, with an increased potential for disease transmission [2,3]. One of its main recommendations is the development of disease surveillance programmes in High Asia to document the presence or prevalence of zoonotic diseases in key wildlife species as a precursor to focused disease monitoring and modelling [3]. Rodents are one of the wildlife taxa specifically highlighted in this context [2].

Rodents are well known globally for their ubiquitous distribution and ability to transmit over 60 zoonotic pathogens, which can have serious effects not only on human health but also the health of livestock and other wildlife [4,5]. In the mountains of Central Asia, people often live in close contact with their livestock [6,7]. Because rodents are attracted by livestock feed and the warmth of human dwellings, and sometimes included in the diet of local people, rodents provide one of the greatest opportunities for disease transmission between wildlife, people and livestock in this region [6,8]. Thus, rodents can be considered as ‘sentinel’ taxa when surveying for zoonotic pathogens of importance in human communities and their livestock [8–10]. From the perspective of wildlife conservation programmes (e.g. the snow leopard *Panthera uncia*) rodents, as a major prey source, may provide a reservoir for cycling important pathogens within ecosystems [11].

Between 2012 and 2015 we surveyed rodents and rodent-like mammals (hereafter ‘rodents’) in the South Gobi region of southern Mongolia for the presence of antibodies to important zoonotic diseases as part of a broader One Health project examining disease transmission potential between humans, domestic dogs, goats and snow leopards [12]. In this context, we identified *Leptospira* spp. Puumala and Seoul hantaviruses, *Toxoplasma gondii and Coxiella burnetii* as the focus of our study, because they are important zoonotic diseases transferred by rodents that can have substantial impacts on the health of humans and wildlife and the viability of livestock herding [13–19]. In addition we surveyed for zoonotic bacteria in rodent ticks and faeces using next generation sequencing; we examined rodent faeces for zoonotic parasites (*Giardia* spp., *Echinoccocus* spp. and *Cryptosporidium* spp.); and finally we tested rodent fleas for the presence of the plague-causing bacteria *Yersinia pestis*. Because of the inherent uncertainties associated with cross-sectional disease surveys in wildlife [20–22], this study is a first step towards generating the necessary pathogen prevalence data required for understanding the risks associated with rodent-borne zoonotic pathogens in High Asian ecosystems. Our goal is that the results of this study will form the basis of future surveys and disease transmission studies to allow effective assessments of infection reservoirs [23], disease dynamics [24] and transmission pathways between hosts [25] to aid in the prevention and management of diseases in this region [2,3].

## Methods

### Study area

**Figure 1.**
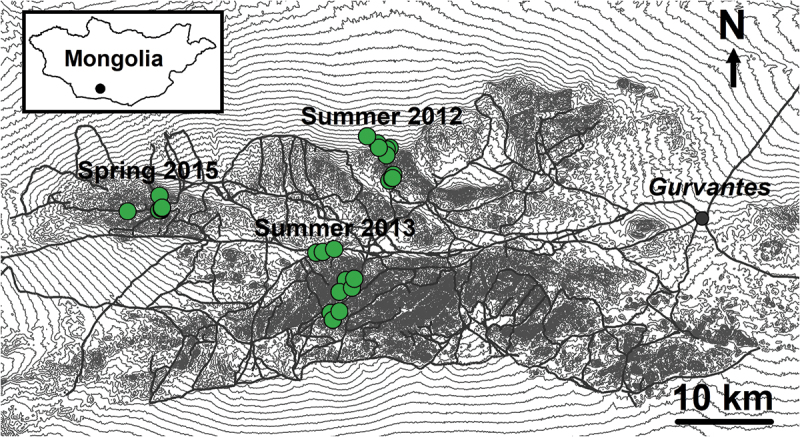
Map of study area in southern Mongolia where green circles show rodent trapping sites in each year. Thin grey lines are 20-meter contour lines and thicker grey lines show small roads that traverse the study area. The town of Gurvantes is shown by the filled grey circle.

The study was conducted in the Tost Mountains in the Gobi Desert (43° N, 100°E) located in the province of Umnogovi in southern Mongolia (Figure 1). The mountains consist of several rugged massifs (altitude 1600–2500 m above sea level), crossed by steep ravines and separated by wider valleys. Vegetation is sparse and consists mainly of short grasses and dwarf shrubs. The climate is windy and dry with <130 mm precipitation annually, of which approximately 70% falls as rain from June–August. The region is cold in winter and hot in summer, with mean daily minimum and maximum temperatures at the eastern end of the study area (1650 m a.s.l.) being −27**°**C and 1**°**C in January, and 11**°**C and 33**°**C in July, respectively. This region is home to an intensively monitored population of snow leopards [11,26] as part of a broader international conservation programme. The human population consists of approximately 90 semi-nomadic families who live in the steppe in the summer and move into the mountains in the winter [27]. Herded livestock, goats and sheep, are active from late morning to dusk and are penned in corrals at night.

### Rodent trapping

Rodent trapping was carried out during three 4-week periods (May and June in 2012 and 2013 and in March and April in 2015) in three subsections of the Tost Mountains (Figure 1). Two sizes of Sherman/Elliot live-traps (SFG Folding Trap 5.08 × 6.35 ×16.51 cm and XLK Folding Trap 7.62 × 9.525 ×30.48 cm) were used. Bait contained a mixture of rolled oats, honey and peanut butter. Eight trap-lines were set in a variety of habitats each year, with 10 traps spaced 20 m apart on each trap-line. Several traps were also set within gers (local dwellings) with the owners’ permission. Traps were set late afternoon and checked early the next morning. Animals were removed from the traps and placed in calico bags and weighed using a Pesola spring balance. Animals were restrained by hand and examined for external wounds, parasites and sex determination before clinical parameters were measured. One ear was notched for future identification, before rodents were released at the point of capture. Ear-notches were stored in 70% ethanol for DNA analyses. Fleas and ticks collected were also stored in 70% ethanol. Trapping was performed under ethics approval A1919 from James Cook University (Australia) and with approval from the Ministry of the Environment in Mongolia.

### Blood, faeces and flea collection

Small rodents (<60 g) were bled via cardiac puncture whereas larger species (>60 g) were bled via cardiac, cephalic or tail veins (collected samples 0.1-1 ml per animal). We did not collect blood from rodents <20 g as blood sampling risked fatally injuring the animal. Blood samples less than 0.5 ml were stored on Advantec Nobuto filter paper strips (Toyo Roshi Kaisha.Ltd, Tokyo Japan 2007). Strips were air-dried and then placed in paper envelopes and stored at room temperature until analyzed. Larger blood samples were placed either into 1.0 ml or 0.5 ml serum separation tubes and spun using a LW Scientific 800 T26 7345 Zip spin solar powered centrifuge. Serum was stored in sterile cryovials at −20.0°C until transport to the laboratory where it was then stored at −80°C. Additional blood was placed in lithium heparin tubes for haematology and biochemical analyses with a hand-held Abaxis I-Stat Analyser (REM Systems Pty, Ltd, North Ryde, Australia). Two cartridges were used per sample, with each cartridge requiring only two drops of blood. Blood smears were fixed directly in the field, stained using haematoxylin and eosin diff kwik stains once back at the laboratory, and subsequently examined microscopically at 40 × and 100 × magnification for any red and white cell abnormalities and haemoparasites. Faeces were collected from the trap or the holding bag and placed in RNAlater (Sigma-Aldrich Pty Ltd Castle Hill NSW 1765 Australia) as a means of preserving them in the field or air dried and stored in paper envelopes at room temperature until analyzed.

### Species identification

Species were identified using a combination of morphological features [28] and DNA extracted from the ear notches and faecal samples. Illumina amplicon sequencing followed a modified Miseq protocol (Metagenomic Sequencing Library Preparation), with sample genomic DNA subjected to PCR amplification targeting a ~ 133-bp fragment of the cytochrome oxydase I gene (COI) using a modified forward primer LepF1 and a modified reverse primer EPT-long-univR (for full details see [29]). The sequences were sorted using a bioinformatic script and compared with sequences available in BOLD databases [30]. Sequences that had a unique best-hit with an identity score greater than or equal to 98% were considered to be positive matches for species identification. For a summary of species caught and sampled see Table 1.

**Table 1.** Summary of the rodent species caught during the study (voles were only identified to the genus level), disaggregated by sex and year of capture.

Rodent Species	2012		2013		2015	
	Female	Male	Female	Male	Female	Male
Midday gerbil (*Meriones meridianus*)	2	4	9	10	0	1
Long-tailed dwarf hamster (*Cricetulus longicaudatus*)	5	7	8	6	7	10
Grey dwarf hamster (*Cricetulus migratorius*)	2	1	6	6	1	5
Kam dwarf hamster (*Cricetulus kamensis*)	5	0	0	0	0	0
Voles (*Alticola spp*.)	0	0	6	3	0	0
Mongolian five-toed jerboa (*Allactaga sibirica*)	4	1	6	5	0	0
Red-cheeked ground squirrel (*Spermophils erythrogenys*)	10	1	8	1	0	0
Mongolian pika (*Ochotona pallasi*)	0	0	2	0	1	0

### Elution of sera from Nobuto strips

Nobuto strips were cut into small pieces and placed in Eppendorf tubes with 200 µl phosphate buffer solution (PBS) if only one side of the strip was saturated with blood or 400 µl of PBS if both sides of the strip were saturated with blood. Nobuto strips were placed in the solution for one hour for the serum to elute. The serum in the tubes were heat-inactivated at 60°C for one hour, centrifuged and the filter papers removed. Remaining serum was then stored at −80°C until analyzed.

### Pathogen identification using ELISA, MAT, NGS and RT-PCR

**1. Leptospira spp**

Serum was initially screened for *Leptospira* antibodies using a qualitative rat *Leptospira* IgG (LS-IgG) ELISA kit, (MBS036971, Mybiosource, San Diego, California, USA) which uses a double antigen sandwich ELISA. Plates were pre-coated with rat *Leptospira* antigen, *L. icterohaemorrhagiae* and *L. grippotyphosa* and the appropriate horseradish peroxidase (HRP) conjugated antigen before adding test samples. The plate results were read at 450 nm on a Thermo Scientific Multiskan FC plate reader as above. Positive samples were sent to the National Veterinary Institute (Sweden) for Microscopic Agglutination Tests (MAT [31]), and tested against the following serovars: Australis, Hebdomadis, Icterohaemorrhagiae, Pomona, Canicola, Grippotyphosa. The antigens used were live cultures of referenced strains. All sera that gave a positive reaction at a 1:100 dilution were further titrated in serial two-fold dilutions to titre endpoint that is 50% agglutination. A titre ≥100 was deemed positive to exposure to *Leptospira*.

**2. Hantaviruses**

Rodent serum samples were tested for antibodies against Puumala (PUUV) and Seoul (SEOV) hantaviruses. A PUUV IgG ELISA was run using the protocol described by Verner-Carlsson et al. [32]. Serum was tested for antibodies to SEOV using an IgG monoclonal antibody (mAb)-capture ELISA [32]. However, due to the various and sometimes high levels of serological cross-reactions among the hantaviruses, no neutralization assays (the only serological assay that is able to detect antibodies at a virus species level) were applied to confirm the ELISA-data. Thus, our results should be regarded as ‘hantavirus seropositives’, or ‘Puumala-like and Seoul-like seropositives’.

**3. Toxoplasma gondii**

Antibodies to *Toxoplasma gondii* were detected using an ABNOVA IgG antibody ELISA kit (ABNOVA, Taipei City 114, Taiwan). This was a human kit, so it was modified by replacing the enzyme conjugate with the appropriate rodent conjugate. The species of rodents were unknown at the time of sample analyses, so test samples were run to determine which enzyme conjugate to use (anti-rat, anti-hamster or anti-mouse) depending on the lineage of the rodent being tested. The plate results were read at 450 nm on a Thermo Scientific Multiskan FC plate reader (Thermo Scientific, Vantaa, Finland). A positive result was recorded when the optical density was greater than the critical cut-off value.

**4. Coxiella burnetii**

Serum was screened for antibodies to *C. burnetii* using an Innovative Diagnostics Q Fever Indirect Multi-species enzyme linked immunosorbent assay ELISA kit (Idvet, 310, rue Louis Pasteur – Grabels-France). The ELISA was performed on the eluted serum samples following the manufacturers’ protocol. The plate results were read at 450 nm on a Thermo Scientific Multiskan FC plate reader (Thermo Scientific, Vantaa, Finland).

**5. Yersinia pestis**

Fleas were tested for *Y. pestis* using an EZ1 machine from Qiagen with a DNA tissue kit. A minimum of five fleas were placed in Eppendorf tubes and crushed with a pestle. The crushed fleas were then lysed in 2 ml Nuclisens lysis buffer (bioMérieux) for one hour. Two hundred microlitres were placed in the DNA extraction machine where each extraction panel consisted of six samples with five microlitres of seal herpes virions added to one sample as an internal extraction control. One hundred microlitres of DNA was eluted from each sample, which was then run by RT-PCR with a specific probe for *Y. pestis*. Fleas were tested in preference to rodent serum, because fleas are the main vectors of *Y. pestis* and seropositive rodents were highly unlikely to be caught because they quickly die after seroconverting.

**6. Bacteria & parasite screening**

In addition to the research of specific pathogens, we also performed a general screening for zoonotic bacteria putatively present on rodents in the studied area. To achieve this goal we used Next Generation Sequencing (NGS), allowing us to screen a large panel of bacteria present in rodent ticks and faeces. Here, the aim was largely complementary to the research on specific pathogens mentioned above. These NGS analyses detect all bacterial genera present, including endosymbionts or opportunists [33]. Total genomic DNA from faeces were subjected to PCR amplification targeting a ~ 142-bp fragment of the 16S rRNA variable regions 5 and 6 (V5-V6) using the primer pair 784F-1061 R originally designed by [34] coupled with the Illumina overhand adapters. Quantified products were then pooled in equimolarity and sent to the GIGA Genomics platform (Ulg) for sequencing on an ILLUMINA MiSeq V2 benchtop sequencer. Faecal samples were screened for *Giardia* spp., *Echinoccocus* spp. and *Cryptosporidium* spp. following the protocols in Ghosh et al. [35] and Chaya and Parija [33]. Six ticks from red-cheeked ground squirrel and long-tailed dwarf hamster were also tested for bacterial genera using the same protocol as for the faecal samples.

### Statistics

We present summarised pathogen prevalence data based on different grouping categories: all animals surveyed, specific rodent taxa and sampling year. These are reported as both raw data observations and derived prevalence estimates from logit-link binomial generalised linear mixed models (GLMMs). We used a GLMM framework for estimating group-level prevalences by fitting the groups to a hierarchical ‘random effect’ structure (see Appendix S1) that modelled group differences around a normal distribution. This approach has the benefit of providing group-level estimates that are conditional on the sample size within the group: i.e. when samples are large the group prevalence estimate will resemble the observed prevalence; however, as sample sizes become smaller, the group estimate increasingly reflects a sample-size weighted compromise between the observed prevalence in that group and the expected prevalence given data from other groups [36]. These estimates were derived using a Bayesian framework using JAGS [37] in R [38] to best incorporate prevalence estimation uncertainties (see Appendix S1).

## Results

### Rodent trapping and field-collections

We collected blood samples from 142 rodent-like mammals (females = 82; males = 60) between May 2012 and April 2015 (Table 1). These animals were identified to seven species and one genera: *Spermophils erythrogenys* (red-cheeked ground squirrel), *Allactaga sibirica* (Mongolian five-toed jerboa), *Meriones meridianus* (midday gerbil), *Cricetulus longicaudatus* (long-tailed dwarf hamster), *C. migratorius* (grey dwarf hamster), *C. kamensis* (Kam dwarf hamster), *Ochotona pallasi* (Pallas’s pika or Mongolian pika) and *Alticola spp*. (voles identified to genus only). For estimating pathogen prevalences, these species and genera were summarised into five taxonomic families to increase sample sizes in each group and subsequently improve the robustness of prevalence estimates: (1) *Muridae* for gerbils (*n* = 26), (2) *Cricetidae* for hamsters and voles (*n* = 77), (3) *Dipodidae* for jerboa (*n* = 16), (4) *Sciuridae* for squirrels (*n* = 20), and (5) *Ochotonidae* for pika (*n* = 3), which is not a rodent but a lagomorph.

### Physical condition, haematology and biochemistry

The animals appeared in good physical health except one lethargic juvenile ground squirrel with a heavy flea burden, and one hamster that was missing an ear. No other external wounds were observed. Twenty-six percent of the females captured were either pregnant or lactating at the time of capture. No adult male red-cheeked ground squirrels were caught. The haematology and biochemistry results from the mammals in this study fell mostly within reference ranges reported for other rodents [39] (see Table S1). One jerboa was slightly anaemic with a haematocrit of 24% (mean haematocrit levels were 42 ± 9.7%) and a long-tailed dwarf hamster had a creatinine level that was off the scale of the analyser. However, both animals were negative for the pathogen antibodies tested.

### Pathogen antibody prevalences

There was considerable variation in the prevalence of antibodies to the different pathogens tested, with antibodies to *Leptospira* being detected in approximately 38% of animals summarised across all years, while antibodies to the hantaviruses were much lower (Puumala ~5.5% of tested animals and Seoul < 1%; Table 2). Interestingly, both the raw data and the prevalence estimates from our modelling show remarkable consistency in the rate of positive detections across the different taxa sampled for each pathogen (Table 2). Here, although there is some variation between taxa, there is no obvious taxonomic group that appears to be more or less likely to carry antibodies to the different pathogens. There was, however, some evidence of variation between sampling years in prevalence estimates; however this yearly variation was not consistent between the different pathogens (e.g. *Leptospira* had its highest prevalence in 2015, while *Toxoplasma* had its lowest in this year; Table 2). There was no evidence that pregnant or lactating females had a higher pathogen seroprevalence to other females, if anything their seroprevalence was lower for some pathogens (reproductive versus non-reproductive female seroprevalence; Leptospirosis: 3/13 versus 15/47; Puumala: 0/15 versus 5/50; Toxoplasma: 4/16 versus 11/48; Coxiella: 0/13 versus 9/39). All fleas collected from 71 rodents were negative for *Y. pestis* by RT-PCR; therefore, rodent sera were not tested for *Y. pestis*.

**Table 2.** Estimates of antibody prevalence for five major pathogens based on samples collected from individual rodents and lagomorphs during three sampling events between 2012 and 2015. Prevalence estimates are presented for each pathogen based on: (1) raw data observations summarised across five taxa (Muridae = gerbils, Cricetidae = hamsters and voles, Dipodidae = jerboa, Sciuridae = squirrels and Ochotonidae = pika); these are shown as the number of individuals that tested positive/the total number of individuals tested, and (2) estimated prevalences for each pathogen as derived from GLMMs and summarised according to taxa (across all years), sampling year (across all taxa), and a global prevalence estimate (across all taxa and years). All estimates are described in terms of the mean ± SD of the posterior probability distribution for that group, with the global estimate including the 95% CIs. Because the estimated prevalences are derived from a modelled distribution they do not precisely describe the raw data, but rather use the data from all groups to adjust their group-level mean estimates relative to the group-level sample sizes (see methods).

		Pathogen			
	Leptospira	Puumala Hantavirus	Seoul Hantavirus	Toxoplasma	Coxiella
Raw data observations					
Muridae	8/25	3/26	0/23	2/25	1/21
Cricetidae	27/67	2/65	0/65	8/65	5/56
Dipodidae	6/15	1/16	1/15	3/14	3/11
Sciuridae	3/12	1/17	0/17	5/18	3/16
Ochotonidae	2/2	0/2	0/3	2/3	1/2
Estimated prevalence					
Taxa					
Muridae	0.35 ± 0.07	0.08 ± 0.04	0.006 ± 0.01	0.12 ± 0.05	0.08 ± 0.04
Cricetidae	0.39 ± 0.05	0.04 ± 0.02	0.005 ± 0.01	0.14 ± 0.04	0.10 ± 0.04
Dipodidae	0.39 ± 0.08	0.06 ± 0.04	0.03 ± 0.03	0.20 ± 0.08	0.19 ± 0.10
Sciuridae	0.33 ± 0.09	0.06 ± 0.04	0.007 ± 0.01	0.23 ± 0.09	0.16 ± 0.07
Ochotonidae	0.47 ± 0.17	0.06 ± 0.07	0.01 ± 0.03	0.33 ± 0.20	0.18 ± 0.13
Sampling year					
2012	0.21 ± 0.07	0.07 ± 0.03	0.017 ± 0.02	0.12 ± 0.05	0.05 ± 0.03
2013	0.41 ± 0.06	0.05 ± 0.02	0.004 ± 0.01	0.20 ± 0.04	0.18 ± 0.05
2015	0.57 ± 0.10	0.03 ± 0.03	0.008 ± 0.01	0.09 ± 0.05	0.11 ± 0.06
Global estimate					
mean ± SE	0.38 ± 0.04	0.055 ± 0.02	0.008 ± 0.01	0.16 ± 0.03	0.12 ± 0.03
95% CIs	0.30–0.47	0.025–0.102	0.001–0.031	0.10–0.23	0.067–0.191

### Next Generation Sequencing (NGS) of rodent ticks and faeces

NGS analyses from 24 ticks resulted in identification of 216 genera of bacteria with 21 of these genera identified as potentially zoonotic. From 39 rodent faecal samples, 250 genera of bacteria were identified with 17 of these genera being potentially zoonotic (Table 3). One tick from a ground squirrel was positive for *Yersinia* sp., but the pathogen could not be identified to species. *Giardia*, *Cryptosporidium* and *Echinococcus* were not identified from any faecal samples.

**Table 3.** Genera of the zoonotic bacteria identified in rodent ticks (*n* = 24; pooled into 8 samples) and rodent faeces (*n* = 39; pooled into 9 samples) where bacterial genera were determined by using NGS methods.

Genus of bacteria	Prevalence in rodent ticks		Prevalence in rodent faeces	
*Aeromonas*	0%	0/8	11%	1/9
*Bacillus*	12%	1/8	11%	1/9
*Bacteroides*	38%	3/8	11%	1/9
*Bartonella*	12%	1/8	11%	1/9
*Brachyspira*	0%	0/8	11%	1/9
*Bordetella*	12%	1/8	0%	0/9
*Burkholderia*	100%	8/8	11%	1/9
*Campylobacter*	0%	0/8	22%	2/9
*Clostridia*	100%	8/8	11%	1/9
*Corynebacterium*	100%	8/8	0%	0/9
*Coxiella*	75%	6/8	0%	0/9
*Enterococcus*	12%	1/8	11%	1/9
*Escherichia/Shigella*	50%	4/8	11%	1/9
*Francisella*	12%	1/8	11%	1/9
*Helicobacter*	12%	1/8	11%	1/9
*Legionella*	50%	4/8	11%	1/9
*Mycobacterium*	25%	2/8	0%	0/9
*Mycoplasma*	0%	0/8	11%	1/9
*Pandoraea*	100%	8/8	0%	0/9
*Pseudomonas*	0%	0/8	11%	1/9
*Rhodococcus*	38%	3/8	0%	0/9
*Rickettsia*	100%	8/8	11%	1/9
*Staphylococcus*	100%	8/8	0%	0/9
*Streptococcus*	63%	5/8	0%	0/9
*Treponema*	12%	1/8	11%	1/9
*Yersinia*	12%	1/8	0%	0/9

## Discussion

The interpretation of disease prevalences from serological data is complicated by many factors that increase both the uncertainty of prevalence estimates (e.g. serological reversion, cross-reactivity, titre threshold levels [20; 21]), and obscure relationships of potential importance (e.g. prevalence dynamics and the relative importance of different pathogens) may be misinterpreted because of the timing of sampling [21,24]. Despite our data being largely cross-sectional (with a longitudinal component in the serology), and being spatially and temporally restricted in their scope, they do provide important baseline data to inform future directions of human-livestock-wildlife disease surveillance, transmission studies and health monitoring in this region [3].

### Rodent serology

There were clear differences in the prevalence estimates between the main pathogens examined using serological antibody testing (Table 2). *Leptospira* spp. prevalence was estimated to be >1/3 of all animals tested, compared to *Toxoplasma* and *Coxiella* (approx. 1/8 animals tested were seropositive), followed by the hantaviruses (Puumala approx. 1/20 and Seoul <1/100). Interestingly, these pathogen-specific differences in prevalence were remarkably consistent between the five taxonomic groups examined (e.g. *Leptospira* 0.33–0.47; *Coxiella* 0.08–0.19; *Puumala hantavirus* 0.04–0.08). This raises the possibility that rodent taxa act as epidemiologically connected populations where some or all of these pathogens are maintained in multi-host reservoirs [25]. In such a system, not all small mammal species need to be direct source populations to be epidemiologically important. Here, a restricted set of small mammal species may act as maintenance populations for the pathogen, and infect other rodent species which may come into contact with people and livestock. Thus, for understanding the relative importance of different rodent species in relation to zoonosis risk, future work will be required to build on our results to identify the role of different species for the maintenance and transmission of pathogens within this multi-host system [23,25].

Despite the relative between-taxa consistency in pathogen prevalences, there were differences between the taxonomic groups and years that require explanation. For *Leptospira* spp. we found that the observed variation in prevalence between taxonomic groups could easily be explained by natural sampling variation and sample size; here the fact that the mammal group with the highest raw data prevalence (100%) and estimated prevalence (0.47) was derived from only two Pika (family *Ochotonidae*) that both tested positive. However, we also need to be mindful that we do not completely discount the possibility of species-level variation in pathogen prevalences. From our data we cannot rule out that Pika are particularly susceptible to *Leptospira*, *Toxoplasma* and *Coxiella* but resistant to infection by the Hantaviruses, despite the observed patterns most likely arising from a small sample size. The same should also be considered for the yearly variation in pathogen prevalences we observed (e.g. Leptospira 0.21–0.57; Puumala-like hantavirus 0.03–0.07). Between-year differences in prevalence may also represent natural sampling variation; however, there are three things to consider. First is that we know pathogen prevalence likely varies between years because of variation in host-pathogen-environment interactions [21]. Second is that our sampling in 2015 was almost 2 months earlier than in 2012–2013, and pathogen dynamics could also be expected to vary during that period in an environment that fluctuates between seasonal extremes. Finally, sampling in each year was undertaken in different areas of the mountains, potentially reflecting local variation in pathogen prevalence across different mammal subpopulations. These potential sources of variation in pathogen prevalence has important implications for future studies. Rather than discounting the apparent effect of year on pathogen prevalence estimates because of spatial and temporal confounds, we should use this information to guide future sampling efforts to examine for potential impacts of these specific effects: i.e. within and between year sampling regimes across a spatial gradient to examine the relative impact of these factors if we think them important in driving pathogen dynamics and zoonosis risk.

Although we detected Puumala-like hantavirus-reactive antibodies in four rodent species and Seoul-like hantavirus-reactive antibodies in one ground squirrel, it is known that hantavirus testing suffers from high cross-reactivity between different hantaviruses [40]. Thus, we can only say with certainty that the animals were for seropositive for hantavirus, but are less certain of the exact type. Gerbils were trapped inside gers and were one of the species that was seropositive to Puumala-like hantavirus, suggesting a potential transmission route from rodents and humans. In Inner Mongolia, south of the study area, haemorrhagic fever with renal syndrome is a significant public health risk caused by Hantaan hantavirus and from the Seoul hantavirus [19]. There are no reports of hantavirus infection in people living in the study area, although this may reflect lack of testing rather than lack of infection. More frequent sampling of the rodents and people in the region and using PCR to test tissue samples is necessary to understand the epidemiology of hantaviruses in this region.

### Faecal and tick-borne bacteria & parasites

Seventeen genera of potentially zoonotic bacteria were detected in the rodent faeces, many of which were also identified in the ticks collected from the rodents. Moreover, many of the bacteria found in the ticks and faeces can cause serious illness in both animals and humans, reinforcing the need for adequate hygiene measures and the removal of rodents from gers. Only a few of these zoonotic bacteria specifically use ticks as vectors, but these include *Coxiella burnetii* and *Francisella tularensis* which are highly infectious and can cause serious illness in both people and livestock [16,41]. Maintenance hosts for the ticks are usually rodents or lagomorphs and spillover hosts can be any mammal [42]. Ticks and fleas are also vectors for *Yersinia* spp. including *Yersinia pestis*, which causes plague [43]. In Mongolia, a number of endemic plague regions have been identified including part of the Umnogovi province where we worked but to the east of the study area (with gerbils and jerboas identified as the main reservoir species [10]). Because both these species occur in our study area, *Y. pestis* should still be considered a probable zoonosis in the Tost region, even if not identified in our study. *Cryptosporidium, Giardia* and *Echinococcus* were not identified in faecal samples; however, the amount of DNA recovered may have been too low to give a positive reading (false negative), or it may have reflected general low burdens of these parasites in the region. These zoonotic parasites have been found in faeces from other rodent species in other areas and can be a significant cause of gastrointestinal illness in people and other animals [44], and should be monitored in future studies.

### From disease surveillance to epidemiological modelling

A valuable first step in understanding the epidemiology of a system begins with the development of conceptual models that suggest linkages between target and source populations and the likely passage of pathogens between species [20]. It is from this foundation that focussed data collection can enable the development of quantitative epidemiological models for predicting and managing disease outbreaks [20,45]. Disease monitoring and knowledge of transmission risks between wildlife, livestock and people in Mongolia and High Asia is limited and scattered, currently hampering the development of conceptual and quantitative epidemiological models. Our study, along with other recent studies surveying infectious and zoonotic diseases in Mongolia (e.g [11,14,16,18,46–49,,,,,,,]), can serve as the baseline patterns of disease incidence and prevalence needed to infer disease transmission between source and target populations in this region [25]. The need for these models is particularly acute, as rapid changes in both local and global factors are accelerating the risks of emerging infectious disease outbreaks in High Asia [2,3]. Thus, while broad survey data as we present in this study are vital elements in the initial understanding of disease ecology and dynamics, future studies need to increasingly focus on specific questions arising from conceptual modelling of these systems to clarify reservoir-target transmission dynamics. It is only then that we will be able to shift our efforts from a reactive response to disease outbreaks, to proactive mitigation strategies based on strong empirical data and quantitative predictive modelling.

## Supplementary Material

Supplemental Material

## Data Availability

Data from the study are attached in the supplementary material.
