# Supplementary material for: The prevalence of rodent-borne zoonotic pathogens in the South Gobi desert region of Mongolia
Source: Infect Ecol Epidemiol. 2023 Oct 19;13(1):2270258. doi: 10.1080/20008686.2023.2270258 (PMC10588514; doi:10.1080/20008686.2023.2270258)
Supplement: Supplemental Material [file ZIEE_A_2270258_SM4407.zip › Supplement_Appendix_S1.docx]

Supplementary Material for the paper:

The prevalence of rodent-borne zoonotic pathogens in the South Gobi Desert region of Mongolia

**Appendix S1**: Model structure of the logit-link binomial generalised linear mixed model (GLMM) for estimating group-level prevalences by fitting the groups to a hierarchical ‘random effect’ structure. Because the observed data are at an individual level (1/0) we use a Bernoulli likelihood distribution. In the model below, ‘tested positive’ is a 1/0 response variable describing if an individual animal was seropositive to a specific pathogen, *p* is the probability parameter being estimated in the model, ‘intercept’ is the grouping-level variable (species or year depending on the analysis), μ is the mean of the distribution describing the variation between the groups, σ is the standard deviation of the distribution describing the variation between the groups, the index ‘i’ refers to the individual observation and the index ‘j' refers to the groups at the grouping-level (j=5 for species, j=3 for year).

Tested Positive _i_  ~ Bernoulli ( *p* _i_ )

logit( *p* _i_ ) = intercept _j_

intercept _j_ ~ Normal ( μ, σ )

μ ~ Normal (0, 100)

σ ~ Uniform (0, 5)
