# Supplementary material for: The prevalence of rodent-borne zoonotic pathogens in the South Gobi desert region of Mongolia
Source: Infect Ecol Epidemiol. 2023 Oct 19;13(1):2270258. doi: 10.1080/20008686.2023.2270258 (PMC10588514; doi:10.1080/20008686.2023.2270258)
Supplement: Supplemental Material [file ZIEE_A_2270258_SM4407.zip › Supplement_Table_S1.docx]

**Table S1**. Haematology and biochemistry values (mean ± SD) for rodents captured in the Tost Mountains from 2012 to 2015. Readers are cautioned that the serum chemistry levels measured in samples eluted from Nobuto strips are likely subject to additional error due to variation in the volume originally collected. We include these data because such baseline haematology and biochemistry values for wild animals may be an important reference for future studies on the physiological health of these species in relation to pathogen load (especially the Siberian jerboa and the red-cheeked ground squirrel having no previously published reference values).

| Rodent species | Na*  mmol/L | K mmol/L | Cl  mmol/L | iCa | TCO_2_ | Glu  mmol/L | Urea mmol/L | Crea µmol/L | Hct  % | Hb  g/dL | AnGap mmol/L |
| --- | --- | --- | --- | --- | --- | --- | --- | --- | --- | --- | --- |
| Ground squirrel (N=5) | 138.4±3.8 | 4.7±1.6 | 106.8±2.6 | 1±0.1 | 23.2±2.2 | 8.3±1.7 | 12±2.6 | 32.8±4 | 43.4±6.3 | 14.8±2.2 | 14.4±2.6 |
| Jerboa  (N=5) | 151±2.3 | 4.7±2.3 | 121±4.0 | 1±0.1 | 15±7.2 | 9.2±3.6 | 8.9±0.9 | 22.8±5.2 | 41.6±9.7 | 14.2±3.3 | 20.8±5.5 |
| Midday gerbil  (N=5) | 154±4.3 | 4.9±2.8 | 125±7.1 | 1.2±0.1 | 12.6±5.1 | 138±3.8 | 11.1±4.1 |  | 42.2±5.0 | 14.3±1.5 |  |
| LT dwarf hamster (N=4) | 143.0 | 6.9 | 128.3 | 1.1 | 11.3 | 7.1 | 14.6 |  | 42.3 | 14.4 | 21.5 |
| Pika  (N=2) | 129.0 | 4.2 | 97.0 | 1.08 | 12.0 | 12.0 | 5.6 | <18 | 39.0 | 13.3 | 26.0 |

*Na = Sodium, K = Potassium , Cl = Chloride, Ca = Calcium, TCO_2 =_ Carbon dioxide, Glu = Glucose, Crea = Creatinine, Hct = Haematocrit, Hb = Haemoglobin, AnGap = Anion gap
